# Functional Polymorphisms of FAS and FASL Gene and Risk of Breast Cancer – Pilot Study of 134 Cases

**DOI:** 10.1371/journal.pone.0053075

**Published:** 2013-01-11

**Authors:** Mohammad Hashemi, Aliakbar Fazaeli, Saeid Ghavami, Ebrahim Eskandari-Nasab, Farshid Arbabi, Mohammad Ali Mashhadi, Mohsen Taheri, Wiem Chaabane, Mayur V. Jain, Marek J. Łos

**Affiliations:** 1 Cellular and Molecular Research Center, Zahedan University of Medical Sciences, Zahedan, Iran; 2 Department of Clinical Biochemistry, School of Medicine, Zahedan University of Medical Science, Zahedan, Iran; 3 Department of Internal Medicine, School of Medicine, Zahedan University of Medical Science, Zahedan, Iran; 4 Biology of Breathing Group, Manitoba Institute of Child Health, Winnipeg, Manitoba, Canada; 5 Department of Physiology, University of Manitoba, Winnipeg, Manitoba, Canada; 6 Genetic of Non Communicable Disease Research Center, Zahedan University of Medical Science, Zahedan, Iran; 7 Department of Clinical and Experimental Medicine, Integrative Regenerative Med. Center (IGEN), Division of Cell Biology, Linköping University, Linköping, Sweden; 8 Department of Biology, Faculty of Sciences, University of Tunis, Tunis, Tunisia; 9 BioApplications Enterprises, Winnipeg, Manitoba, Canada; The Chinese University of Hong Kong, Hong Kong

## Abstract

Fas/Fas ligand (FasL) system is one of the key apoptotic signaling entities in the extrinsic apoptotic pathway. De-regulation of this pathway, i.e. by mutations may prevent the immune system from the removal of newly-formed tumor cells, and thus lead to tumor formation. The present study investigated the association between −1377 G/A (rs2234767) and −670 A/G (rs1800682) polymorphisms in Fas as well as single nucleotide polymorphisms INV2nt −124 A/G (rs5030772) and −844 C/T (rs763110) in FasL in a sample of Iranian patients with breast cancer. This case-control study was done on 134 breast cancer patients and 152 normal women. Genomic DNA was extracted from whole blood samples. The polymorphisms were determined by using tetra-ARMS-PCR method. There was no significant difference in the genotype distribution of *FAS* rs2234767 polymorphism between cases and controls. *FAS* rs1800682, *FASL* rs5030772, and *FASL* rs763110 genotypes showed significant associations with an increasing risk of breast cancer (odds ratio OR = 3.18, P = 0.019; OR = 5.08, P = 0.012; OR = 2.40, P = 0.024, respectively). In conclusion, *FAS* rs2234767 was not associated with breast cancer risk. Though, *FAS* rs1800682, *FASL* rs5030772, and *FASL* rs763110 polymorphisms were associated with the risk of breast cancer in the examined population.

## Introduction

Breast cancer is the main cause of cancer-related death among women in western countries, with the sporadic form of the disease constituting more than 90% of all breast cancers [1], [2]. Breast cancer is also one of the most frequent malignancies among Iranian women [3], [4]. In addition to environmental factors and body weight, genetic invariability affects not only the chance of cancer development but also its aggressiveness, therapy response and overall prognosis [5], [6], [7]. Apoptosis is one of physiological forms of cell death that plays an important role in maintaining tissue homeostasis. Abnormal regulation of apoptosis contributes to the pathogenesis of cancer [8], [9]. The acquired capability to oppose apoptotic stimuli is one of the main characteristics of a malignant cell. Alterations of apoptotic pathways are key events in the development of variety of human diseases including cancer [10], [11].

The *FAS*/*FASL* signaling system activates a major extrinsic cell death pathway, that is of particular importance for the regulation of acquired immune response, maintaining immuno-privileged sites and fulfils other regulatory functions [12], [13]. Fas (TNFRSF6/CD95/APO-1) is a cell surface receptor, expressed in a variety of tissues [14]. FasL, also known as TNFSF6 or CD95L, is a member of the tumor necrosis factor superfamily and the natural ligand to Fas [15]. The Fas interaction with the FasL may trigger the death signal cascade, and eventually the cell expressing FAS would die. Studies have shown that decreased expression of Fas and/or elevated expression of FasL could be detected in many kinds of human tumors [16], [17].

The *FAS*/*FASL* system may have two opposite effects on cancer. The expression of FAS on tumor cells, if downstream signaling pathways are functional, may assist FAS-triggered killing of tumors by immune-effector cells [15], [18]. The expression of FASL on tumor cells may repel specific antitumor immune response, thus turning tumor into an immuno-privileged site [19], [20]. The role anticancer-drug-induced *FAS*/*FASL* system in tumor-cell killing has been championed by some [21], it is currently however often disregarded as experimental artifact [22], [23].

Two polymorphisms have been identified in the FAS promoter region: one in the silencer region, G to A substitution at nucleotide position −1377 (rs2234767), and the other in the enhancer region, A to G substitution at nucleotide position −670 (rs1800682). These two polymorphisms are located within the stimulatory protein-1 (Sp1) and the signal transducers and activators of transcription 1 (STAT1) transcription factor binding sites, respectively [24], [25]. Because these sequence variations in the *FAS* gene promoter region may influence Fas expression and deregulate cell death signaling, they could contribute to carcinogenesis [25]. The human *FASL* gene is located on chromosome 1q23, consists of four exons spanning ∼8 kb, and encodes 281amino acids [26]. There are two reported polymorphisms: C to T changes at nucleotide position −844 (FASL-844 C/T, rs763110) in the promoter region [27] and A to G change at nucleotide position −124 of intron 2 (FASL INV2nt −124 A/G, rs5030772). FASL −844 C/T is located in a putative binding motif for a transcription factor, CAAT/enhancer-binding protein h, and the −844 C allele may increase basal expression of FASL compared with the −844 T allele [27], suggesting that the FASL −844 C/T polymorphism may influence *FASL* expression and FasL mediated signaling, and ultimately, the susceptibility to cancer. So far, the functional relevance of the *FASL* IVS2nt −124 A/G polymorphism has not been reported.

Numerous studies investigated the role of *FAS* and *FASL* gene polymorphisms in the etiology of various cancers including cervix, breast, bladder, lung, prostate, head and neck and esophagus [25], [28], [29]. However, the role of *FAS* and *FASL* gene polymorphisms in breast cancer has not been conclusively established. Thus, the present study, that assesses the association between −1377 G/A and −670 A/G polymorphisms in *FAS* as well as single nucleotide polymorphisms −844 C/T and INV2nt −124 A/G in *FASL* adds an important information about the role of this death pathway in breast cancer.

## Materials and Methods

### Cancer patients and controls

This case control study was performed in 134 patients with histologically confirmed breast cancer and in 152 population based healthy women who participate in a screening project for metabolic syndrome with no history of cancer. The local Ethical Committee of Zahedan University of Medical Sciences approved the study protocol and written informed consent was obtained from all subjects. Two ml of venous blood drawn from each subject and genomic DNA was extracted from peripheral blood as described previously [30].

### T-ARMS-PCR assay

Tetra-amplification refractory mutation system–polymerase chain reaction (T-ARMS-PCR) is a rapid and simple technique for detection of single nucleotide polymorphism [30], [31], [32]. In the present study we designed T-ARMS-PCR assay for the detection FAS and FASL polymorphisms. The primers used are listed in Table 1. Two allele-specific (inner) primers have been designed in opposed directions and, in combination with the common primers, can simultaneously amplify both the wild-type and the mutant alleles in a single-tube PCR (see result section for schematic depiction).

**Table 1:** Tetra-ARMS-PCR primers and conditions for Fas, −1377 A/G (rs2234767), −670 A/G (rs1800682) and FasL Ivs2nt 124 A/G (rs5030772), FASL −844 C/T (rs763110).

Primers	Sequence (5′→3′)	Annealing temp. (°C)
Fas −1377 A/G (rs2234767)		
FO	CCTTCCCTCACACCCCTTTTCCTTCC	64
RO	CTTTGGCATCGTCCACCAAGCTCTG	
FI	AGTGTGTGCACAAGGCTGGCCCA	
RI	TTAGTGCCATGAGGAAGACCCTGTGC	
Fas −670 A/G (rs1800682)		
FO	GGGGCTATGCGATTTGGCTTAAGTTGTT	62
RO	GTAGTTCAACCTGGGAAGTTGGGGAGGT	
FI	TTTTTCATATGGTTAACTGTCCATTCCCGG	
RI	GCAACATGAGAGGCTCACAGACGGTT	
FasL Ivs2nt 124 A/G (rs5030772)		
FO	GGTCTTCTTGGATTAGTCACCCAACTT	57
RO	CACTTTCCTCAGCTCCTTTTTTTCAG	
FI	CTGCAGTTCAGACCTACATGATTAGTCTG	
RI	TTAAAACCGTAAATGGCAACAGTCTAAAAT	
FASL −844 C/T (rs763110)		
FO	TGCAGTTAACTACGATAGCACCACTGCA	63
RO	AGGAGGAAAACATCTGTTGCCATCATCT	
FI	GGCAAACAATGAAAATGAAAACATGGC	
RI	AACCCACAGAGCTGCTTTGTATTGCA	

PCR was performed using commercially available PCR premix (AccuPower PCR PreMix, BIONEER, Daejeon, Korea) according to the manufacturer's instructions. For detection of polymorphisms of FAS −670 A/G (rs1800682) and −1377 A/G (rs2234767), 1 µL template DNA (∼100 ng/µL), 1 µL of each primer (10 pmol/µL), and 15 µL DNase-free water were added into a 20 µL PCR tube containing the AccuPower PCR PreMix. The same PCR condition was used for detection of FASL (rs763110), except that 0.6 µL of each primer and 16.6 µL water were added. For FASL (rs5030772), 1 µL template DNA, 1 µL of outer primer, 0.5 µL of inner primer, 0.5 µL of DMSO and 15.5 µL was added.

PCR cycling was performed at 95°C for 5 min, followed by 30 cycles of a denaturation for 30 s at 95°C, annealing for 30 s at different temperatures (Table 1), extension s at 72°C (30 s for rs1800682 and rs2234767; 40 s for rs5030772 and rs763110) and a final extension for 10 min at 72°C in a thermocycler (Corbett research, Australia). The amplified products were separated by electrophoresis on a 2% agarose gel containing 0.5 µg/ml ethidium bromide.

The genotyping analysis was randomly repeated for 10% of samples for confirmation for quality control and the results were 100% concordant. To confirm the genotyping results, selected PCR-amplified DNA samples (n = 3, respectively, for each genotype) were examined by DNA sequencing and the results determined by T-ARMS-PCR were concordant with those determined by sequencing (see the result section for more details).

### Real-time RT–PCR (qRT–PCR) assay

We investigated the expression levels of FAS in 10 tumor samples and 16 adjacent normal tissue breast normal tissues. Briefly, total RNA was extracted from the tissues using TRIzol reagent. Then complementary DNA (cDNA) synthesis from total RNA was catalyzed in a final volume of 25 µL by cDNA synthesis kit (Bioneer, K-2045, Korea) according to the manufacturer's protocol. Expressions for the Fas and the housekeeping GAPDH genes were investigated using real-time quantitative PCR (qRT-PCR) (Applied Biosystems). FAS (F: 5′-TGAAGGACATGGCTTAGAAGTG-3′, R: 5′-GGTGCAAGGGTCACAGTGTT-3′) and GAPDH primers (F: 5′-GAAGGTGAAGGTCGGAGTC-3′, R: 5′- GAAGATGGTGATGGGATTTC-3′) were used [33]. Samples were assayed in a 25 µL reaction mixture including 2 µL of cDNA, 12.5 µL of 2× Master Mix (Fermentas, Cat No. K0221), 1 µL of each specific primer (10 µM), and 8.5 µL of RNase-free water. qRT-PCR reactions were performed with an initial incubation at 95°C for 10 minutes followed by 40 cycles at 95°C for 15 seconds and 60°C for 60 seconds.

### Statistical analysis

The statistical analysis of the data was performed using the SPSS 18.0 software. Genotypes and alleles were compared between groups by use of χ2 test. The strength of the association between the polymorphisms and cancer risk was measured by odds ratios (ORs) with 95% confidence intervals (CIs). Binary logistic regression was used for all analysis variables to estimate risk as odds ratio (OR) with 95% confidence intervals (CIs) using age as covariate. According to our findings, sample power was calculated for FAS and FASL polymorphisms by comparison of each genotype with the sum of other related genotypes (Table 2) at each polymorphic region by using STATA 10 software and is shown in table 3.

**Table 2:** Clinical and pathological characteristics of breast carcinoma patients.

Characteristic	Number
Stage	
I	28
II	63
III	26
IV	15
Unknown	2
Grade	
I	23
II	72
III	16
IV	18
Unknown	5
Estrogen Receptor	
Positive	70
Negative	61
Unknown	3
Progesterone Receptor	
Positive	69
Negative	58
Unknown	7
HER2	
Positive	51
Negative	80
Unknown	3
Histology	
Ductal carcinoma	101
Lobular carcinoma	7
Other	22

**Table 3:** Frequency distribution of Fas rs2234767, rs1800682 and FasL rs5030772, rs763110 gene polymorphisms in normal and breast cancer individuals.

Polymorphisms	Breast cancer n (%)	Normal n (%)	*OR (95% CI)	p-value	Study power %
FAS					
−1377A/G (rs2234767)					
AA	8 (6.0)	11 (7.2)	1.00	-	4
AG	106 (79.1)	115 (75.7)	1.49 (0.48–4.65)	0.429	8
GG	20 (14.9)	26 (17.1)	1.24 (0.34–4.49)	0.747	5
Alleles					
A	122 (45.5)	140 (46.0)	1.00	-	3
G	146 (54.5)	164 (54.0)	1.03 (0.74–1.42)	0.877	3
−670 A/G (rs1800682)					
AA	55 (41.0)	63 (41.4)	1.00	-	3
AG	55 (41.0)	78 (51.3)	1.04 (0.56–1.92)	0.901	37
GG	24 (18.0)	11 (7.3)	3.18 (1.21–8.33)	0.019	73
Alleles					
A	165 (61.6)	204 (67.1)	1.00	-	25
G	103 (38.4)	100 (32.9)	1.27 (0.90–1.79)	0.189	25
FASL					
Ivs2nt 124 A/G (rs5030772)					
AA	77 (57.4)	92 (60.5)	1.00	-	6
AG	40 (29.9)	55 (36.2)	0.87 (0.47–1.59)	0.650	17
GG	17 (12.7)	5 (3.3)	5.08 (1.04–18.22)	0.012	78
Alleles					
A	194 (72.4)	239 (78.6)	1.00	-	50
G	74 (27.6)	65 (21.4)	1.40 (0.95–2.06)	0.096	50
−844 C/T (rs763110)					
CC	42 (31.3)	62 (40.8)	1.00	-	34
CT	51 (38.1)	64 (42.1)	1.17 (0.58–2.36)	0.663	10
TT	41 (30.6)	26 (17.1)	2.40 (1.12–5.14)	0.024	73
Alleles					
C	135 (50.4)	188 (61.8)	1.00	-	76
T	133 (49.6)	116 (38.2)	1.61 (1.14–2.24)	0.007	76

* Adjusted for age, age at menarche, menopausal status, body mass index (BMI).

## Results

**Figure 1:**
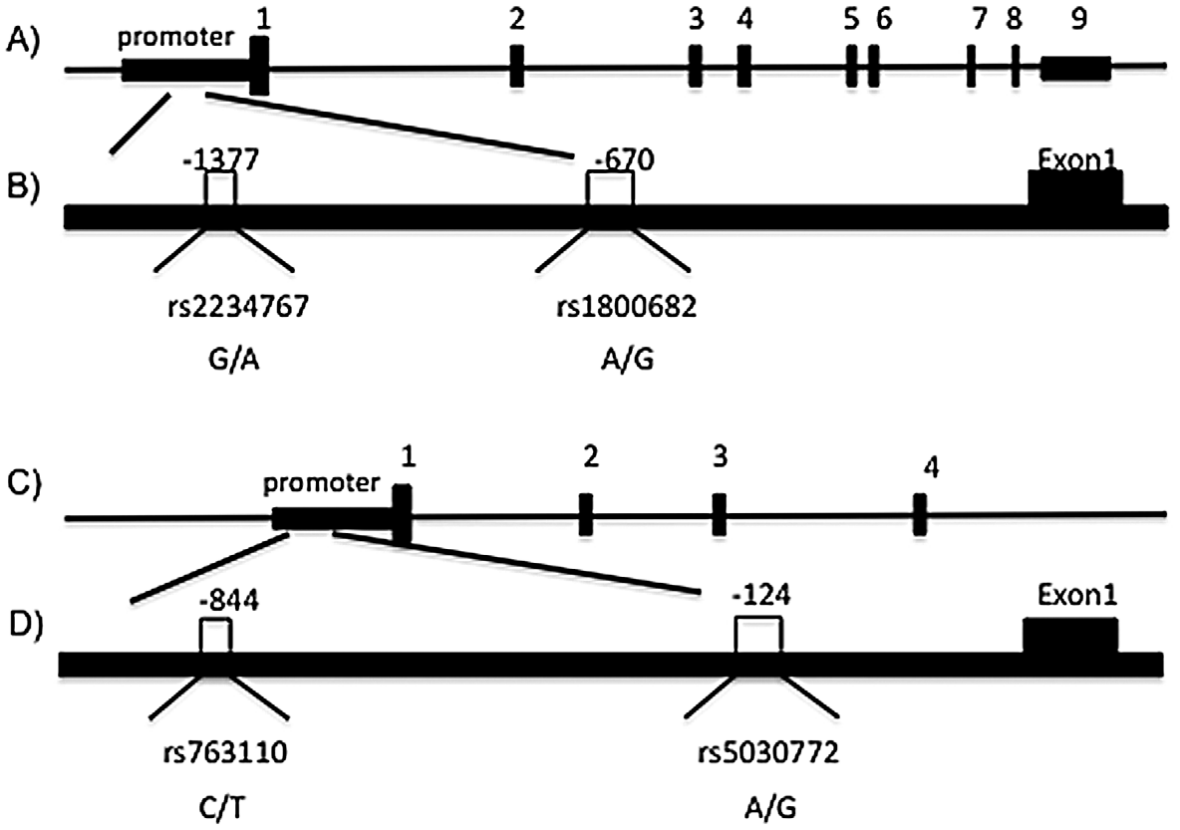
Maps of the human Fas and FasL genes with polymorphism positions indicated. (A) Map of the human Fas gene. Exons 1–9 are numbered and represented by black boxes. (B) position of the single nucleotid variations within the core promoter of the Fas gene, a G>A polymorphism at position −1377 and a A>G polymorphism at position 670. (C) Map of the human Fas Ligand gene. Exons 1–4 are numbered and represented by black boxes. (D) Position of single nucleotide polymorphisms (SNPs) within the Fas Ligand promoter, a C>T substitution at position −844 and a A<G substitution at position −124.

**Figure 2:**
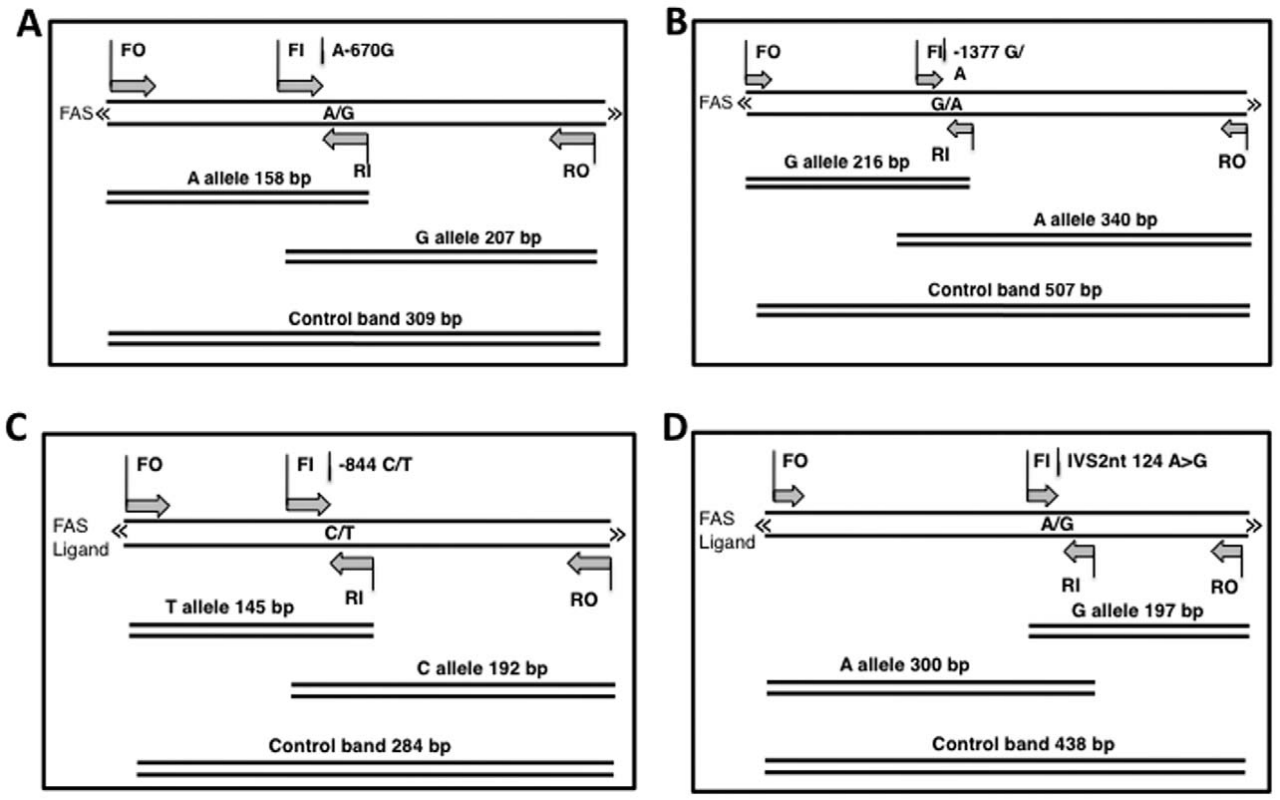
Schematic representation of Tetra-Primer Amplification Refractory Mutation System. T-ARMS-PCR was used for the detection of SNPs of Fas rs1800682 (**A**), Fas rs2234727 (**B**), FasL rs763110 (**C**) and FasL rs5030772 (**D**). Two forward and two reverse specific primers are used to produce three potential products. Product sizes were 158 bp for A allele, 207 bp for G allele, and 309 bp for two outer primers (control band) for Fas rs1800682 (**A**). Product sizes were 216 bp for G allele, 340 bp for A allele, and 507 bp for control band for Fas rs2234727 (**B**). Product sizes were 145 bp for T allele, 192 bp for C allele, and 284 bp for control band for FasL rs763110 (**C**). Product sizes were 197 bp for G allele, 300 bp for A allele, and 438 bp for control band for FasL rs5030772 (**D**).

**Figure 3:**
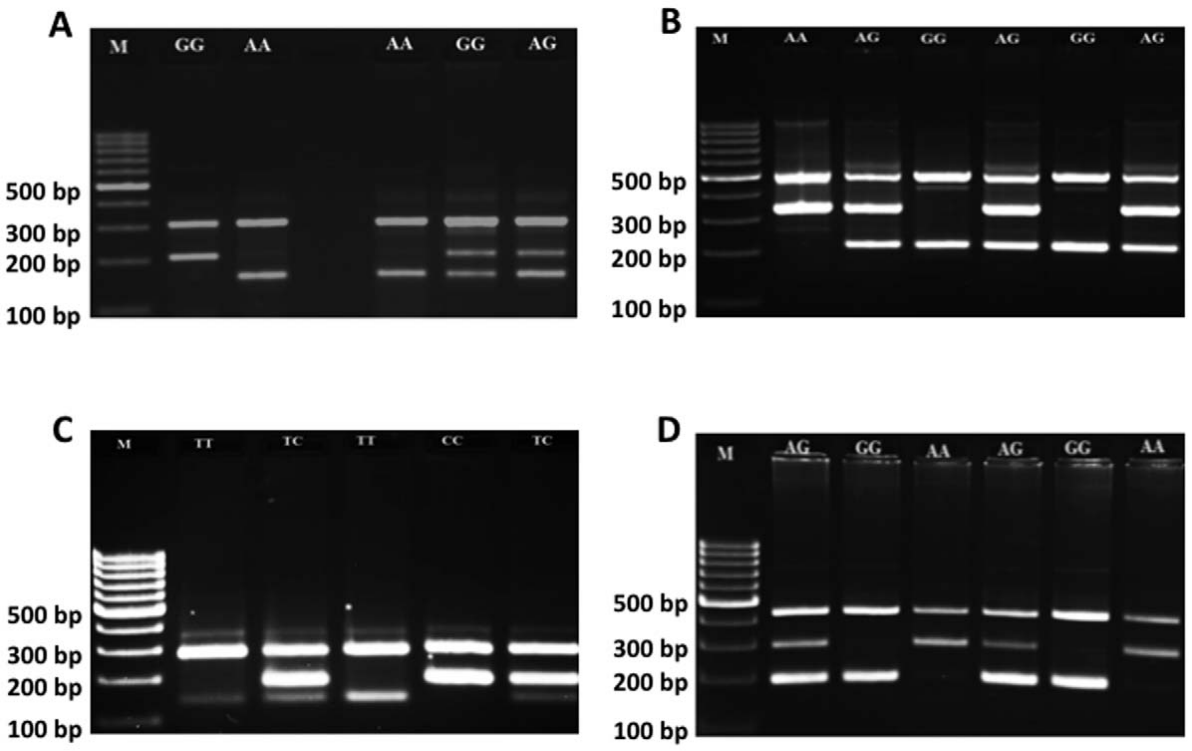
Electrophoresis pattern of tetra-ARMS-PCR for detection of polymorphisms. Agarose gel electrophoresis was used to detect band-pattern of tetra-ARMS-PCR for Fas rs1800682 (**A**), Fas rs2234727 (**B**), FasL rs763110 (**C**), and FasL rs5030772 (**D**). M = DNA marker.

**Figure 4:**
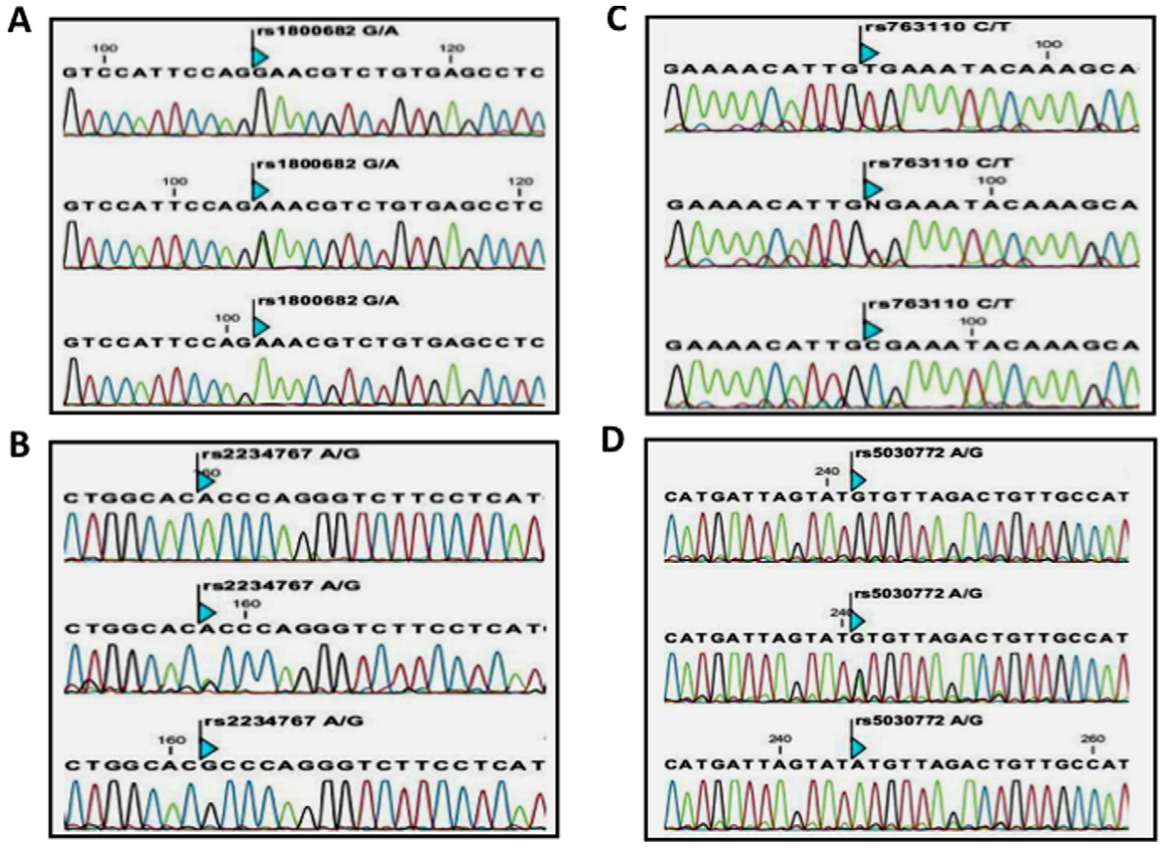
Examples of DNA sequencing results of Fas and FasL prlymorphisms. DNA sequencing results of Fas rs1800682 (**A**), Fas rs2234727 (**B**), FasL rs763110 (**C**) and FasL rs5030772 (**D**) for tetra-ARMS-PCR results depicted in figure 3 are shown.

The study group consists of 134 histopathologically confirmed female cases with breast cancer (age; 47.97±13.27 years), and 152 healthy females (age; 43.53±13.52 years). The clinicopathologic characteristics of patients are summarized in Table 2. The present study examined the association between −1377 G/A and −670 A/G polymorphisms in *FAS* as well as single nucleotide polymorphisms −844 C/T and INV2nt −124 A/G in *FASL* (Fig. 1). Should any of the studied polymorphisms be significantly associated with ant of the type of breast cancer, it would provide important indirect information about the role of death pathway in breast cancer. The study was performed applying Tetra-amplification refractory mutation system–polymerase chain reaction. The stretches of genes targeted for amplification are depicted in figure 2 and the corresponding primers used for the T-ARMS-PCR are shown in Table 1. The amplified products were separated by electrophoresis on a 2% agarose gel containing 0.5 µg/ml ethidium bromide (please see figure 3 as an example). For quality-control purposes, the genotyping analysis was randomly repeated for 10% of samples for confirmation for quality control and the results were 100% concordant. To confirm the genotyping results, selected PCR-amplified DNA samples (n = 3, respectively, for each genotype) were examined by DNA sequencing and the results determined by T-ARMS-PCR (Fig. 3) were concordant with those determined by sequencing (Fig. 4). Table 3 summarizes the genotype and allele frequencies of the examined polymorphisms in the *FAS* gene promoter region (−1377 G/A, rs2234767; and −670 A/G, rs1800682), as well as two in the FASL gene (−844 C/T in the promoter region, rs763110; and −124A/G in the second intron, rs5030772). Significances of associations (p-values) of those alleles with breast cancer risk are also shown in Table 3 (second-from the right column).

The current study does not demonstrate a significant difference in *FAS* −1377A/G genotype frequencies between patients and controls (χ^2^ = 0.49, p = 0.78). Hence no association was observed between −1377A/G polymorphism of *FAS* and risk of breast cancer (Table 3). On the other hand the genotype distribution of the *FAS*–670A/G polymorphism was significantly different between patients and control groups (χ2 = 8.25, p = 0.016). There was a significant association between the *FAS* −670 A/G polymorphism and breast cancer risk and the −670 GG genotype is a risk factor for susceptibility to breast cancer (OR = 3.181; 95% CI = 1.21–8.33; p = 0.019).

There was a significant difference between breast cancer subjects and controls in the distribution of *FAS*L Ivs2nt 124 A/G (rs5030772) genotypes (χ2 = 9.15, p = 0.010) and the GG genotype exhibited significant association with the risk of breast cancer (OR = 5.08; 95% CI = 1.04–18.022; p = 0.012). A significant difference was identified in the distribution of *FASL* −844 C/T genotype between patients and controls (χ2 = 7.57, p = 0.022) and the TT genotype showed an increased risk for breast cancer (OR = 2.40; 95% CI = 1.12–5.14; p = 0.024).

Genotype distribution of *FAS*-1377A/G polymorphism in case and control are not consistent with Hardy–Weinberg equilibrium (HWE). The distribution of *FAS* −670 A/G genotype did not deviate from the HWE in case patients or control subjects. While, genotype distribution of *FASL* Ivs2nt 124 A/G and −844 C/T deviate from the HWE in case group. Furthermore, haplotypes analysis was performed (Tables 3 and 4). The results showed that rs2234767A/rs1800682A/rs5030772A/rs763110T, rs2234767G/rs1800682G/rs5030772A/rs763110T and rs2234767A/rs1800682A/rs5030772G/rs763110T haplotypes decreased the risk of breast cancer (OR = 0.18, 95%CI = 0.04–0.88, p = 0.04, OR = 0.03, 95%CI = 0.00–0.43, p<0.01, and OR = 0.04, 95%CI = 0.00–0.60, p = 0.02, respectively) in comparison with rs2234767G/rs1800682A/rs5030772A/rs763110C haplotype.

**Table 4:** Haplotype frequencies of FAS (rs2234767, rs1800682) and FasL (rs5030772, rs763110) genes polymorphisms in normal and breast cancer subjects.

rs2234767	rs1800682	rs5030772	rs763110	Case	Control	OR (95%CI)	P
G	A	A	C	0.1032	0.1949	1.00	-
A	A	A	C	0.1294	0.1699	1.42 (0.28–7.09)	0.67
A	G	A	T	0.1034	0.1054	2.57 (0.42–15.60)	0.30
G	A	A	T	0.0694	0.1353	0.69 (0.20–2.36)	0.55
G	G	A	C	0.1125	0.0755	0.56 (0.12–2.60)	0.46
A	A	A	T	0.0879	0.0346	0.18 (0.04–0.88)	0.04
G	A	G	C	0.0961	0.0115	0.88 (0.13–5.96)	0.90
A	G	A	C	0.0354	0.0783	0.28 (0.04–2.12)	0.22
G	A	G	T	0.0219	0.0591	6.28 (0.51–77.32)	0.15
G	G	A	T	0.0330	0.0195	0.03 (0.00–0.43)	<0.01
A	A	G	T	0.0488	0.0604	0.04 (0.00–0.60)	0.02
A	A	G	C	0.0184	0.0149	0.11 (0.01–2.07)	0.14
G	G	G	T	0.0745	0.0081	0.03 (0.00–1.02)	0.05
G	G	G	C	0.0269	0.0084	0.14 (0.01–2.41)	0.18
A	G	G	C	0	0.0242	2.51 (0.12–54.32)	0.56

The expression level of *Fas* was determined in breast cancer and normal tissues by qRT-PCR using the 2^−ΔΔCT^ method. The results showed that the expression levels of *FAS* mRNA were not significantly different between breast cancer and normal tissues (P = 0.588).

## Discussion

Breast tumor cells frequently down-regulate FAS and/or up-regulate FASL expression [34], [35], [36], [37]. Death receptor activation initiates extrinsic apoptotic pathways, which in some cell types may be sufficient to carry apoptosis, whereas in others it also branches towards the intrinsic/mitochondrial pathway, via caspase-8-dependent Bid-cleavage [38], [39], [40]. Numerous polymorphism sites have been identified to influence the development of breast cancer [2], [7]. In the present study we found lack of association between FAS expression and breast cancer. In normal breast epithelium, Fas protein is expressed constitutively, while in primary breast cancer, its expression was found to be less uniform [41]. It has been reported that the lack of Fas in the primary tumor was associated with perilymphatic fat infiltration and metastasis either to the regional lymph nodes or to the bones [42], [43].

Several previous researches have focused on the association between different gene polymorphisms within cell death pathways and the risk of developing malignancies and developing cancers. FAS/FASL polymorphisms commands major attention as it may play different, context-dependent roles in cancer [44], [45]; 1) apoptosis promotion by FasL on T-lymphocytes in Fas-expressing cancer cells, this mechanism could play an important responsibility in cell mediated cytotoxic reactions against malignant cells. 2) malignant cells could escape immune system expression of FasL, thus repelling Fas-expressing immune-effector cells, hence preventing the immune system from recognizing mutated tumor cells (tumor as immuno-privileged site). 3) FasL expressed on T cells, may not only aids them to kill target (cancer) cells, but it was also associated with an enhanced rate of activation-induced cell death in T cells [46]. Various correlations have been drawn from genetic polymorphisms in the death pathway genes *FAS* and *FASL* and the risk of cancer development [47], [48], [49], [50], [51], [52].

In the present study, we investigate whether the *FAS*-1377 G/A, *FAS*-670 A/G, *FASL*-844 T/C and *FASL* Ivs2nt 124 A/G polymorphism in cell death pathway genes were associated with the risk of the development of breast cancer in a sample of Iranian population (south east of Iran). We found that the *FAS*-1377 G/A did not affect the risk of breast cancer, while *FAS* −670 G/A, *FASL* Ivs2nt 124 A/G and *FASL* −844 C/T gene polymorphisms are risk factors for this disease in our study population. Previous researches have also addressed FAS/FASL polymorphisms in breast cancer but in some of these studies the results only partially corroborated with our findings. Crew et al., have reported no association between breast cancer and *FAS* −1377 G/A, *FAS* −670 G/A and *FASL* −844C/T polymorphisms [53]. On the other hand, Zhang et al have found a significant association between *FAS* −1377G/A and *FASL*-844T/C gene polymorphisms and risk of breast cancer, but they reported no association between *FAS* −670 G/A and breast cancer risk [46]. We observed a significant association between breast cancer risk and *FAS* −670 G/A genotype, thus our findings show partly-different association pattern that the above mentioned ones. Moreover, they observed a significantly increased breast cancer risk associated with the *FAS* −1377G/A genotype, but we report lack of such association. They reported decreased risk associated with *FASL* −844TT (OR, 0.66; 95% CI, 0.43–1.00) genotype, but we found increased risk with mentioned genotype (OR, 2.40; 95% CI, 1.12–5.14). Contrary to our results, Krippl et al., in a study of 500 breast cancer patients and 500 controls in a Caucasian population in Austria, reported a significant association between *FAS* −1377G/A and increased risk of breast cancer, but they found no associations with *FAS* −670G/A and FASL −844C/T [54]. The *FASL* −844 C/T has a considerable impact on the promoter activity of the *FASL* gene in an in vitro assay system, because the polymorphism affects a binding affinity for the transcription factor C/EBP. A significantly higher basal expression of *FasL* is associated with the *FasL* −844 C allele compared with the T allele [27]. Hence, the *FASL* −844 C allele, which drives a higher expression of FASL in T cells, was associated with an enhanced rate of activation-induced cell death in T cells [46]. It has been reported that *FASL* −844 T allele has a possible protective effect on cancer risk [55]. Although the exact mechanism for this inverse association in our study was not clear, other polymorphisms of FASL may also play a role. There is no clear explanation for deviation from HWE in our population. The possible reason may be due to genetic drift.

We recognize some limitations of the present study: (*i*) we have no data on some known risk factors (e.g., family history, previous benign conditions, oral contraceptive or hormone therapy use, etc.), (*ii*) a relatively small sample size. Nevertheless, we believe that the presented here data provides an important input into the debate regarding the clinical relevance of investigated polymorphisms.

Our work is a valuable addition to the ongoing discussion on the influence of various polymorphisms within the signaling molecules pertaining to cell death pathways, and cancer formation. The so far only partial overlapping of conclusions reached from our data analysis as compared to previously published studies (please see above) may reflect differences in genetic background between the studied populations, and variations in external (i.e. environmental), or other (i.e. obesity) factors that influence studied populations [5], [8], [56]. As cell death pathways are ubiquitous, active in most cells in eucaryotes, our manuscript provides additional voice in the discussion on the role of Fas/FasL system on the clinical outcome of breast- and other cancers. One has to keep in mind that although apoptosis is the dominant, fastest cell death program, autophagy appears to play increasingly important role as an accessory cell death mechanism [57], [58], [59].
